# Whole-Exome Sequencing of Discordant Monozygotic Twins for Congenital Scoliosis: A Family Case Study

**DOI:** 10.3390/genes16101220

**Published:** 2025-10-15

**Authors:** Diana Samarkhanova, Madina Seidualy, Ulykbek Kairov, Nurbek Nadirov, Maxat Zhabagin

**Affiliations:** 1 National Center for Biotechnology, Astana 010000, Kazakhstan; ms.madise95@gmail.com (M.S.); mzhabagin@gmail.com (M.Z.); 2 Center for Life Sciences, National Laboratory Astana, Nazarbayev University, Astana 010000, Kazakhstan; ulykbek.kairov@nu.edu.kz; 3 Department of Orthopedics, Mother and Child Health Center, University Medical Center, Astana 010000, Kazakhstan

**Keywords:** congenital scoliosis, monozygotic twins, whole-exome sequencing, de novo variants, vertebral malformations

## Abstract

**Background/Objectives**: Congenital scoliosis (CS) is a developmental disorder characterized by abnormal vertebral development during embryogenesis. Despite the identification of genes involved in vertebral development, the underlying genetic causes of CS remain largely unknown. Monozygotic (MZ) twins discordant for CS offer a unique opportunity to explore de novo or postzygotic causes. This exploratory case study aimed to investigate potential causative variants underlying CS using whole-exome sequencing (WES). **Methods**: We performed WES on a Kazakhstani family with MZ twins discordant for congenital scoliosis. Variant prioritization included homozygous mutation analysis in the affected twin, family-based comparisons via de novo, autosomal recessive, and autosomal dominant models, and cross-referencing with variants previously implicated in spinal deformities. **Results**: Key findings include potential associations of the *STOX1* (storkhead box 1), *HOXD8* (homeobox D8), and *C1QTNF9* (C1q- and TNF-related 9) genes with congenital scoliosis. However, subsequent validation revealed low read depth and strand bias. Notably, no unique variants were detected in genes previously known to cause CS. **Conclusions**: The first WES analysis of CS-discordant twins from a single family highlights the feasibility of a combined family-based and twin-comparative analytical pipeline. Our results provide new insights into the genetic architecture of CS and establish a foundation for future twin studies to elucidate the genetic basis of rare developmental disorders.

## 1. Introduction

Congenital scoliosis (CS) is a rare spinal deformity affecting 0.5–1 per 1000 births and is characterized by a disturbance in the formation of the vertebrae during early embryonic development [1]. This condition affects the quality of life of patients, and diagnosis and treatment remain difficult. CS arises from failures in vertebral formation or segmentation, leading to various spinal abnormalities such as wedge-shaped vertebrae, hemivertebrae, butterfly vertebrae, and block vertebrae [2]. Resulting anomalies can progress during childhood and substantially impair quality of life, making diagnosis and treatment challenging. The etiology of CS remains incompletely understood. Its development is believed to involve a complex interplay of genetic predispositions and environmental influences [3]. In certain cases, congenital scoliosis has been linked to genetic syndromes, such as Klippel-Feil syndrome (KFS) and spondylocostal dysostosis [4].

Surgery is the primary and most effective treatment for CS, typically performed in early childhood. Resection of the posterior hemivertebra with transpedicular instruments is currently a widespread surgical method used for the treatment of CS [5]. However, some complications, such as hardware destabilization or abdominal pseudohernia, have been reported after surgery [6,7]. Newer approaches, such as vertebral column manipulation (VCM) systems aim to improve surgical precision and safety in patients with vertebral malformations [8].

While surgery remains the main method of scoliosis treatment, a deeper understanding of the genetic basis of CS is needed to improve diagnosis and selection of therapeutic strategies. At the molecular level, congenital spinal deformities (CSDs) are characterized by disturbed signaling pathways during somitogenesis and vertebral patterning. A recent review summarized the signaling pathways, including the Wnt, Notch, Hedgehog, BMP, and TGF-β pathways that regulate vertebral formation during embryogenesis [9]. Disrupted pathways are associated with the vertebral malformations observed in CS [10].

Advances in genetic technologies, particularly sequencing, have made it possible to determine the genetic basis for the pathogenesis of congenital diseases associated with vertebral malformations. A comprehensive study summarized data and identified 118 genes associated with vertebral malformations [4]. Several genes, such as *TBX6*, *FBN1*, *PTK7*, *SOX9*, *TBXT*, and others, have been identified as key genes responsible for the development of congenital scoliosis [11,12,13,14,15]. Despite advances in understanding the genetics of congenital scoliosis, we still have a limited understanding of the molecular mechanisms of this disease. The main reason is that the genetic basis of congenital scoliosis is likely to be more complex than assumed.

To address the challenges underlying the complex nature of CS, the study of monozygotic (MZ) twins provides a powerful opportunity to investigate the genetic basis of congenital anomalies [16]. Phenotypic differences can be explained by environmental factors, epigenetic factors, and postzygotic mutations. Advances in sequencing have revealed that the discordance may arise from single-nucleotide variants (SNVs), copy number variations (CNVs), indels, and postzygotic mitotic recombination [17,18]. A recent study of MZ twins discordant for adolescent idiopathic scoliosis, via whole genome bisulfite sequencing revealed that the MAPK and PI3K-Akt signaling pathways may contribute to this phenotypic difference [19].

Building upon this foundation, our exploratory single-family case study provides a comprehensive whole-exome sequencing analysis of MZ twins discordant for CS. Through systematic analysis of multiple inheritance models combined with comprehensive evaluation of established CSD gene panels, we aimed to identify genetic variants responsible for CS development and generate hypotheses for future functional and multi-omics studies.

## 2. Materials and Methods

### 2.1. Study Participants

The study included a Kazakhstani family comprising eight members: parents, four siblings, and MZ twins. One twin was radiographically diagnosed with CS characterized by left-sided hemivertebrae at the L2 vertebral level. The remaining family members had no clinical diagnosis of CS. Peripheral blood samples were collected from all participants for subsequent genetic analysis. The study was conducted in accordance with the principles of the Declaration of Helsinki and was approved by the local ethical committee at the LLP “National Center for Biotechnology” (NCB), Astana, Kazakhstan. Written informed consent was obtained from all participants or their legal guardians.

### 2.2. Whole Exome Sequencing

Genomic DNA was extracted from peripheral blood samples using standard protocols. Exome capture was performed via the VAHTS Target Capture Core Exome Panel (Vazyme Biotech Co., Ltd., Nanjing, China). Paired-end libraries (insert size, ~350 bp) were prepared using the VAHTS Universal Plus DNA Library Prep Kit for Illumina (Vazyme Biotech Co., Ltd., Nanjing, China). Quality control of the libraries was performed on a Qsep400 (BiOptic Inc, New Taipei City, Taiwan). Sequencing was conducted on a Genolab M (GeneMind Biosciences Co., Ltd., Shenzhen, China). Sequencing set V 2.0 (FCH 300 cycles) with 500 million reads per flow cell. High-quality sequencing achieved a mean coverage >150× across all samples with >94% of the targets covering ≥20×, ensuring reliable variant detection (Table 1). The coverage consistency between the twins was confirmed before analysis.

### 2.3. Alignment, Variant Calling and Annotation

The raw reads were aligned to the human reference genome (hg19/GRCh37) and variant calling was performed using the DRAGEN Bio-IT Platform (version 07.021.624.3) in joint genotyping mode, generating VCFv4.2-compliant files. Variant annotation was performed using ANNOVAR (release 2022Aug02). Filter-based annotation incorporated ClinVar (version 20220320) and dbNSFP (v4.2a). Population allele frequencies were referenced from gnomAD v2.1.1 and ExAC 0.3. Variants with MAF > 1% in population databases and synonymous variants were excluded; only high-confidence variants marked as ‘PASS’ by the DRAGEN pipeline were retained for downstream analysis.

### 2.4. Variant Filtering

Annotated variants were filtered in three stages. First, we selected protein-altering variants, including nonsynonymous SNVs, stopgain, stoploss, frameshift insertions, and frameshift deletions at exonic or splicing sites. Second, we applied two levels of pathogenicity filtering. Liberal filtering prioritized variants predicted to be deleterious by at least one in silico tool (SIFT, PolyPhen-2 HDIV, or MutationTaster). Strict filtering applied additional quantitative thresholds of CADD > 20 and a REVEL ≥ 0.5 to prioritize variants with strong predicted deleterious impact.

### 2.5. Family-Based Analysis

Variants were analyzed under three inheritance models. In the de novo model, we considered variants that were present in the affected twin and absent in the unaffected twin, parents, and siblings. For the autosomal recessive model, we identified variants that were homozygous (1/1) in the affected twin and heterozygous (0/1) in both parents, while excluding those that were homozygous in the unaffected twin and siblings. The autosomal dominant model required heterozygous variants (0/1) in the affected twin, and homozygous variants (0/0) in the unaffected twin, parents, and siblings. Variants consistent with incomplete penetrance were considered if one parent were heterozygous. Comparisons were performed using bcftools (v1.17) and custom filtering scripts in Python (v3.13.0), and the code used for family-based analyses is available upon request.

### 2.6. Comparison of Established Gene Panels

Comprehensive lists of genes associated with congenital spinal deformities were curated from recent literature [4]. Systematic analysis of all variants within these gene panels was conducted across all family members, with comparison of genotype patterns between discordant twins for each established gene.

### 2.7. Variant Validation and Structural Analysis

Candidate variants were visually inspected in BAM files using Integrative Genomics Viewer (IGV) to verify read support and eliminate potential artifacts. Structural modeling of candidates was performed using the AlphaFold2-predicted model visualized in PyMOL, with functional effects evaluated by DynaMut2.

## 3. Results

### 3.1. Quality Metrics of Whole-Exome Sequencing

High-quality sequencing was obtained for all eight family members, with mean coverage >150×. Uniformity of coverage exceeded 94% across all samples, ensuring reliable variant detection (Table 1).

### 3.2. Variant Filtering and Pedigree Structure

**Figure 1:**
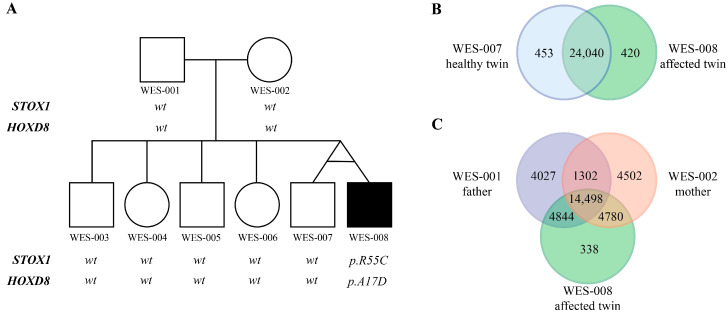
Genetic analysis of MZ twins discordant for CS. (**A**) Family pedigree; the affected twin (WES-008) with L2 hemivertebrae is indicated. (**B**) Venn diagram of variants in discordant twins showing high concordance (98.2% shared). (**C**) Three-way Venn diagram showing variant inheritance patterns from parents to affected twin.

**Figure 2:**
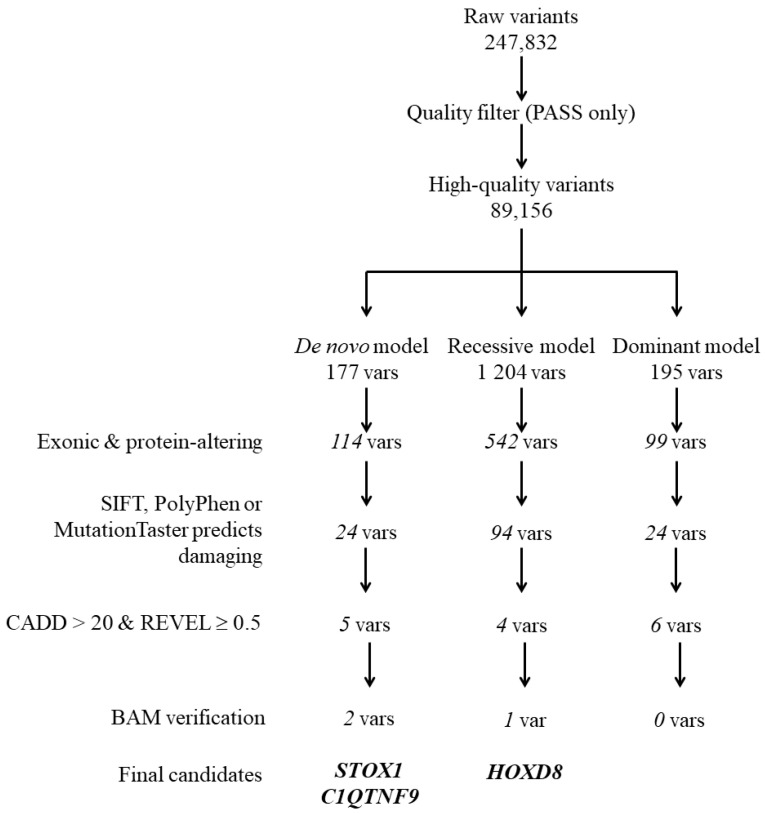
Variant filtering workflow. Sequential filtering of 247,832 variants using functional, pathogenicity and inheritance model criteria. Liberal filtering retained variants predicted as damaging by at least one in silico tool, while strict filtering required CADD > 20 and REVEL ≥ 0.5. *STOX1* and *C1QTNF9* emerged as candidate genes, whereas *HOXD8* required additional validation due to low coverage.

Genetic analysis of the MZ twins discordant for CS is summarized in Figure 1. Systematic filtering of raw 247,832 variants is illustrated in Figure 2; inheritance model analyses reduced the dataset to a limited set of candidate genes.

### 3.3. De Novo Model Analysis

Family-based filtering for de novo variants identified 222 variants potentially unique to the affected twin. Among the exonic variants, 100 nonsynonymous SNVs (87.7%), 10 frameshift insertions (8.8%), and 4 frameshift deletions (3.5%) were detected. The application of liberal pathogenicity filtering, where at least one of these tools, SIFT, PolyPhen2, or MutationTaster, predicts the variant to be damaging, reduces the candidate set to 24 variants. Strict filtering using the CADD > 20 and REVEL ≥ 0.5 criteria resulted in 5 preliminary candidates, which were further supported by manual inspection (Table 2).

### 3.4. Autosomal Recessive Model Analysis

The autosomal recessive model analysis initially identified 1204 candidates, 542 after functional filtering and 94 after liberal pathogenicity filtering. The strict filtering criteria identified 4 recessive candidates including *CPT2*, *LRP2*, *ERCC6L2*, and *NXPE1* (Table 3). However, manual verification revealed that these variants were also homozygous (1/1) in the unaffected twin, eliminating them as causative factors.

### 3.5. Autosomal Dominant Model Analysis

Autosomal dominant model analysis revealed 195 candidates, reduced to 99 after functional filtering and 24 after liberal pathogenicity filtering. Dominant inheritance screening identified 6 potential candidates meeting the filtering criteria, including *FOXD4L1*, *HS6ST1*, *FOXD4L5*, *SVIL*, and *STOX1* (Table 4). Although *HOXD8* variant did not meet the strict CADD (>20) and REVEL (≥0.5) thresholds, it was retained as a candidate based on manual BAM verification and biological relevance. Among these genes, *HOXD8* and *STOX1* presented the most promising patterns on the basis of manual BAM verification.

### 3.6. CSD Gene Panel Analysis

Genes that were previously found to be associated with congenital spinal deformities were compared with our dataset. We manually evaluated 126 unique genes associated with congenital scoliosis, Klippel-Feil syndrome (KFS), and other congenital spinal deformities (CSDs) mentioned in the literature (Table S1). Our analysis included 19 KFS-associated genes, 24 CS-associated genes, and 83 genes linked to other CSDs. Despite this comprehensive coverage of established developmental pathways, all of the analyzed genes were identical genotypes between discordant twins, as confirmed by manual BAM verification.

All major signaling pathways linked to vertebral development such as the BMP pathway, the Notch signaling pathway, which includes genes such as *DLL3*, *JAG1*, and *NOTCH2*, and the Wnt signaling pathway, which includes *FZD6*, *DACT1*, and *DISP2*, were covered in this analysis. Additionally, transcription factors (*TBX6*, *MESP2*, and *PAX3*), structural proteins (*FBN1*, *COL5A1*), multiple collagen subtypes, and planar cell polarity genes (*VANGL1*, *VANGL2*, *SCRIB*, and *CELSR1*) were completely concordant between the MZ twins.

### 3.7. Homozygous Variants

Analysis of homozygous variants in the affected twin yielded 9586 initial candidates, which were reduced to 4276 after filtering by mutation type at the exonic sites and 388 after liberal pathogenicity filtering. Strict filtering criteria identified 9 high-confidence candidates including *CPT2*, *LRP2*, *AMACR*, *DMXL1*, *FOXD4*, *ERCC6L2*, *NXPE1*, *KRT6C*, and *C1QTNF9*. However, manual verification revealed that these variants, except *C1QTNF9*, were also homozygous in the unaffected twin, eliminating them as causative factors.

### 3.8. Protein Structure Modeling and Predicted Functional Impact

To further evaluate the structural and functional relevance of candidate variants, in silico protein modeling was performed for *STOX1* (p.R55C), *HOXD8* (p.A17D), and *C1QTNF9* (p.G143V) genes using AlphaFold2-predicted structures visualized in PyMOL. DynaMut2 was applied to estimate the effects of each amino acid substitution on local stability and conformation.

**Figure 3:**
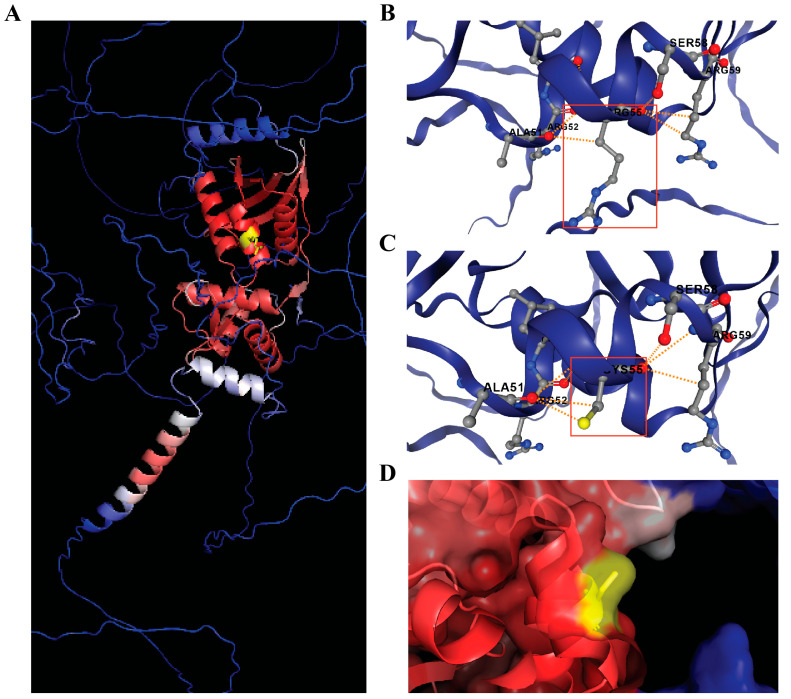
STOX1 protein structure modeling. (**A**) Predicted 3D structure of STOX1 (UniProt Q6ZVD7) obtained from AlphaFold and visualized in PyMOL. The protein is colored by confidence (pLDDT, blue = high > 90, red = low < 70) and the mutation site (Arg55) is highlighted in yellow. (**B**) Wild-type environment around Arg55 predicted by DynaMut2, showing interactions of Arg55 (highlighted by the red box) with surrounding residues Ala51, Arg52, and Ser58. (**C**) Mutant model (Cys55) predicted by DynaMut2, with the same local region highlighted by the red box. Predicted stability effect of p.R55C substitution according to DynaMut2 (ΔΔG = +0.4 kcal/mol). The cysteine substitution introduces an additional polar contact with Ala55 via its sulfur atom (yellow), resulting in three polar interactions instead of two in the wild type, while maintaining the overall backbone conformation. (**D**) Surface view of the mutant STOX1 structure in PyMOL, highlighting the solvent-exposed position of Cys55.

Residue R55 of the STOX1 protein is located N-terminally to the winged-helix DNA-binding domain. The Arg-to-Cys substitution introduces a smaller polar residue at a solvent-exposed site (Figure 3D), predicted to be slightly stabilizing. Although not directly within the DNA-binding domain, the alteration could modulate protein stability.

**Figure 4:**
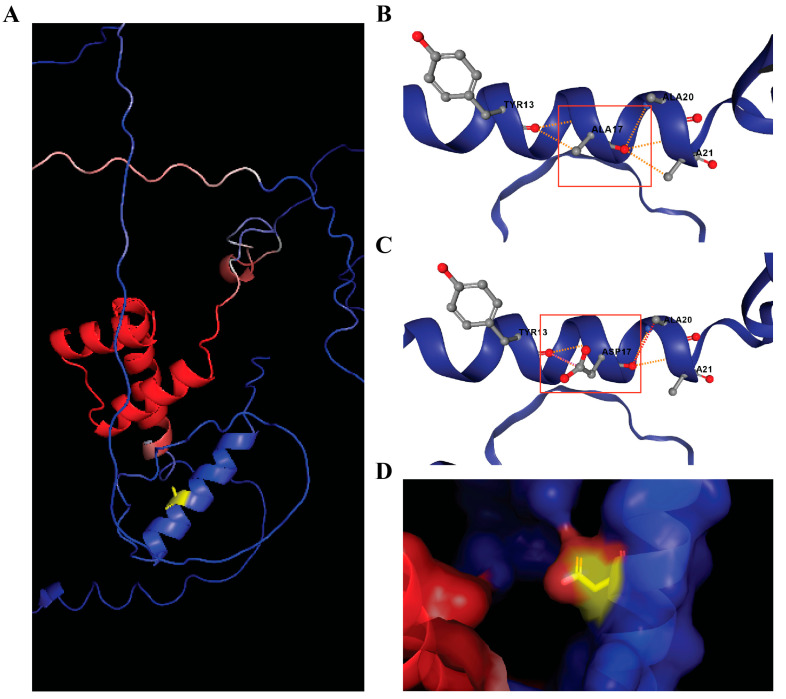
HOXD8 protein structure modeling. (**A**) Predicted 3D structure of HOXD8 (UniProt P13378) obtained from AlphaFold and visualized in PyMOL. The protein is colored by confidence (pLDDT, blue = high > 90, red = low < 70) and the mutation site (Ala17) is highlighted in yellow. (**B**) Wild-type environment around Ala17 predicted by DynaMut2, showing interactions of Ala17 with surrounding residues (region highlighted by red box). (**C**) Mutant Asp17 conformation predicted by DynaMut2, with the same local region highlighted to enable direct comparison. The substitution introduces an additional polar contact to Tyr13. Predicted stability effect of p.A17D is ΔΔG = +0.01 kcal/mol, which is a minimal stability change. (**D**) Surface view of the mutant HOXD8 structure in PyMOL, partially exposed position near the N-terminal helix.

Residue A17 of the HOXD8 protein lies in the flexible N-terminal tail preceding the homeobox domain. The Ala-to-Asp substitution introduces a negatively charged side chain (Figure 4D) but is predicted to have minimal energetic effect. Given the low sequencing depth and borderline pathogenicity scores, this variant is considered biologically plausible but technically uncertain.

**Figure 5:**
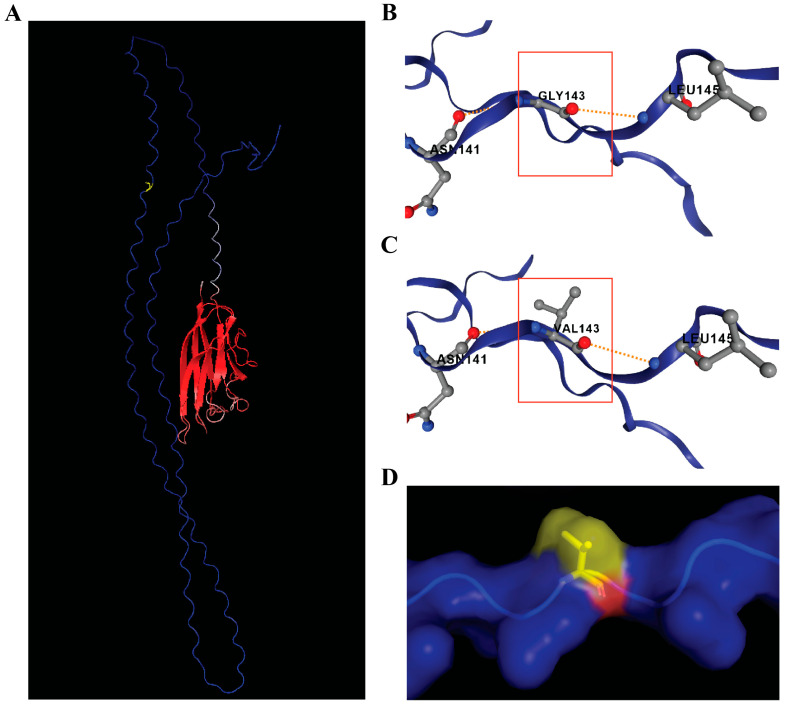
C1QTNF9 protein structure modeling. (**A**) Predicted 3D structure of C1QTNF9 (UniProt P0C862) obtained from AlphaFold and visualized in PyMOL. The protein is colored by confidence (pLDDT, blue = high > 90, red = low < 70) and the mutation site (Gly143) is highlighted in yellow. (**B**) Wild-type environment around Gly143 predicted by DynaMut2 (region highlighted by red box). (**C**) Mutant Val143 conformation predicted by DynaMut2, showing a bulkier side chain with a potential steric hindrance to neighboring residues (red box). Although the visible polar contact with Asn141 and Leu145 are maintained, substitution of glycine with the bulkier valine likely introduces local packing strain and reduced backbone flexibility. Predicted stability effect of p.A17D is ΔΔG = −1.18 kcal/mol (destabilizing). (**D**) Surface view of the mutant C1QTNF9 structure in PyMOL.

G143 of the C1QTNF9 protein resides within the collagen-like region. Replacement of glycine with valine introduces additional steric bulk that may disrupt triple-helix geometry and oligomerization (Figure 5D). Although DynaMut2 predicted a destabilizing effect of p.G143V, the low read depth in WES data indicates that this variant is likely a false positive.

## 4. Discussion

High-quality exome sequencing was achieved for all eight family members, with an average coverage depth across all samples of 159.3× and over 94.9% of the target regions achieved ≥20× coverage.

This study represents the first WES analysis of a family with MZ twins discordant for CS (Figure 1A). Although exploratory, it provides a comprehensive analytical pipeline, combining de novo, autosomal recessive, and autosomal dominant inheritance, supported by manual BAM/IGV verification. We conducted single family-based analysis and provided novel insights into CS genetics and the challenges of twin-based genomic studies (Figure 2).

A total of 5 high-confidence de novo variants were identified in the affected twin (Table 2). These 5 gene candidates were manually verified using their BAM files. Manual BAM inspection clarified that *FOXD4L1*, *FOXD4L5*, and *SVIL* variants were present in unaffected family members and thus unlikely to be causal. Regarding the *STOX1* gene (chr10:70587543), manual BAM analysis confirmed that the heterozygous variant of this gene was present only in the affected twin, whereas other unaffected family members showed a homozygous reference genotype. The read depth was approximately 23–50 reads per sample. With respect to the *C1QTNF9* gene (chr13:24895332), VCF files indicated a homozygous variant (1/1) in the affected twin with no-calls (./.) in other family members. However, BAM analysis revealed limited read coverage (18–28 reads) and only 1/19 reads supporting the variant allele in WES-008, suggesting possible false positive calling. Subsequent IGV visualization revealed inconsistent representation and strand bias, leading to reclassification of both *STOX1* and *C1QTNF9* variants as technical artifacts.

On the basis of our research, the *STOX1* gene was initially considered a candidate, showing twin discordance on manual BAM verification. The *STOX1* gene, a storkhead box 1 gene, encodes a transcription factor involved in cell proliferation and differentiation, the overexpression of which can lead to preeclapsia [20]. The identified variant (chr10:70587543:C>T, p.R55C) affects a highly conserved arginine residue in the DNA-binding domain of this storkhead box transcription factor. While STOX1 protein has been studied primarily in placental biology, its roles in cellular stress responses, developmental timing, and cell cycle regulation during embryogenesis make it a plausible CS candidate [21]. In silico predictions consistently classify p.R55C substitution as damaging (CADD = 26.4, REVEL = 0.535, SIFT = deleterious, PolyPhen-2 = probably damaging), supporting its pathogenic potential. Structural modeling suggested a solvent-exposed position potentially influencing nuclear interactions (Figure 3). However, subsequent orthogonal inspection in IGV revealed low total coverage, strand bias, and 9% alternate allele fraction, indicating the variant was a technical artifact rather than a true de novo event.

Autosomal recessive and dominant models provided additional variants, but manual verification was essential. The autosomal recessive analysis initially suggested *CPT2*, *LRP2*, *ERCC6L2*, and *NXPE1*, yet all were also homozygous in the unaffected twin (Table 3). Autosomal dominant model analysis identified 6 variants, of which only 2 genes, *HOXD8* and *STOX1*, were selected after BAM analysis (Table 4). The analysis of the *HOXD8* gene (chr2:176995144) revealed a heterozygous variant in the affected twin. *HOXD8* gene was of interest because of its role in developmental patterning, but it did not meet the strict filtering thresholds (CADD = 15.3, REVEL = 0.462) and exhibited low read depth. The *HOXD8* variant (pA17D) lies in the N-terminal region preceding the homeobox domain (Figure 4). We therefore interpret *HOXD8* variant (p.A17D) as a biologically plausible but technically uncertain candidate.

*C1QTNF9*, a C1q and tumor necrosis factor-related gene, appeared as a homozygous variant (1/1) in the affected twin with no-calls in all other family members according to VCF analysis. This gene is predicted to be part of the collagen trimer as annotated in the NCBI Gene Database [22]. To evaluate the potential structural consequences of the p.G143V substitution, in silico modeling was performed (Figure 5). However, manual BAM and IGV analysis revealed limited read coverage and only a single alternate read, suggesting possible technical artifacts in variant calling. While the predicted involvement of *C1QTNF9* gene in collagen networks and extracellular matrix organization could be relevant to vertebral development, this specific variant was not supported by IGV analysis. 

The most transformative finding of this study was the identical patterns between MZ twins in 126 genes that were previously found to be associated with CSDs. Congenital scoliosis is largely characterized by mutations in developmental genes such as *TBX6*, *DLL3*, and *MESP2* and components of the Notch, Wnt, and HOX pathways [4]. In contrast, our comprehensive analysis revealed that while variants were present in 69 of the 126 established CSD genes across family members, every single variant demonstrated concordant inheritance with discordant twins. This finding reflects a challenge to current genetic models, as identical genotypes in established CS genes cannot explain the phenotypic differences between twins. Therefore, there is evidence suggesting that CS pathogenesis may lie outside the coding regions of the genome, for example, through epigenetic modifications, environmental factors, or other postzygotic mechanisms that are not captured by standard exome sequencing [23]. Similarly to previous WES studies of discordant MZ twins with microtia-atresia, our findings confirm that coding sequence differences between twins are exceedingly rare [24]. Therefore, post-zygotic mosaicism or epigenetic modulation likely represents the main source of phenotypic discordance.

Somatic mosaicism arises when post-zygotic mutations occur during early embryonic cell divisions, leading to genetically distinct cell populations within an individual. Such events have been reported in multiple MZ twin studies and may underlie discordant phenotypes in identical genomes [25]. In the context of vertebral development, a mutation emerging during somitogenesis could become unevenly distributed among developing somites, affecting only one twin. Because conventional WES captures only systemic variants, low-frequency mosaic variants may remain undetected.

A study of MZ twins with adolescent idiopathic scoliosis (AIS) showed that twins with different degrees of curvature had differentially methylated CpG sites and genes, including *TBX1*, *PAX3*, and *LBX1*, previously described in association with AIS [26]. More gene hypermethylation was detected in twins with high degrees of deformity, and functional analysis revealed pathways associated with skeletal morphogenesis, muscle function, neurotransmission and key signaling cascades such as cAMP, Wnt, and prolactin. These data support the multifactorial nature of the disease, in which epigenetic changes can be both markers of curve progression and potential therapeutic targets.

Our study has several limitations that should be acknowledged. The analysis was conducted on a single family and needs to be replicated on additional discordant twin pairs to establish generalizability. Technical coverage limitations in some genomic regions could miss relevant variants, although our systematic approach minimizes this concern. Additionally, our focus on protein-coding variants through exome sequencing may miss regulatory or structural variants contributing to CS pathogenesis.

Further research could provide greater scientific value by expanding the sample to include more pairs of discordant monozygotic twins, which would increase the statistical significance of the observed differences. In addition, exome sequencing alone may not be sufficient to fully understand the molecular mechanisms underlying phenotypes. Since the genetic identity of twins does not always explain the differences in the manifestation of the disease, it would be better to supplement the analysis with epigenetic profiling methods, including the assessment of DNA methylation, histone modifications, and chromatin structure. Such an integrated approach can help identify postgenomic regulatory factors that play a key role in the pathogenesis of diseases, and thereby expand our understanding of the nature of congenital malformations [27]. Multiomics integration combining genomic, transcriptomic, epigenomic, and proteomic data will be essential for fully characterizing the complex mechanisms underlying CS pathogenesis.

## 5. Conclusions

Whole exome sequencing of a family with MZ twins discordant for congenital scoliosis revealed a small set of candidate variants. Although initial BAM verification suggested *STOX1* as the most promising de novo candidate, subsequent IGV visualization did not confidently support this finding. The *C1QTNF9* variant likewise showed very limited read coverage and was considered a technical artifact, while the *HOXD8* variant remained a biologically plausible but technically uncertain candidate. Importantly, all previously reported CSD genes demonstrated identical inheritance patterns between twins, emphasizing that coding variants in known genes cannot explain phenotypic discordance. As an exploratory single-family case study, these results should be interpreted as hypothesis-generating. These findings underscore the need to explore non-coding variation, epigenetic mechanisms, and postzygotic events to fully understand the genetic architecture of CS. Future studies including additional discordant twin pairs and multi-omics will be essential to validate and expand upon these observations.

## Figures and Tables

**Table 1:** Quality metrics of whole-exome sequencing of all eight family members.

Sample	Average Alignment Coverage over Target Region	Coverage ≥ 20×, %	Coverage ≥ 50×, %	Aligned Reads	Uniformity of Coverage (>0.2 × Mean), %
WES-001	168.62	95.04	92.67	75,343,495	94.18
WES-002	185.68	95.13	93.2	83,246,593	94.13
WES-003	171.22	95	92.74	75,932,971	94.11
WES-004	158.2	94.86	92.27	70,000,230	94.08
WES-005	140.09	94.87	91.12	61,970,997	94.13
WES-006	138.88	94.69	91.04	61,009,330	94.04
WES-007	152.59	94.95	91.75	67,074,657	94.12
WES-008	158.84	94.99	92.24	70,330,963	94.2
Average	159.265	94.94	92.13	70,613,654.4	94.12

Key: WES-001—father, WES-002—mother, WES-003–WES-006—unaffected siblings, WES-007—unaffected twin, WES-008—affected twin.

**Table 2:** High-confidence de novo candidate variants.

Gene	Chr	Exon	cDNA Change	Protein Change	refGene	CADD	REVEL	SIFT	PolyPhen2	Mutation Taster
*FOXD4L1*	2	1	NM_01218:c.C433T	p.R145C	Nonsynonymous SNV	23.2	0.666	D (0.008)	B (0.208)	D (1.0)
*FOXD4L5*	9	1	NM_001126334:c.G512A	p.R171H	Nonsynonymous SNV	21.8	0.567	D (0)	D (1.0)	D (1.0)
*SVIL*	10	20	NM_003174:c.T2843G	p.L948R	Nonsynonymous SNV	24.4	0.5	D (0.001)	D (0.991)	D (0.995)
*STOX1*	10	1	NM_001130159:c.C163T	p.R55C	Nonsynonymous SNV	26.4	0.535	D (0.001)	D (1.0)	D (0.998)
*C1QTNF9*	13	4	NM_001303138:c.G428T	p.G143V	Nonsynonymous SNV	23.0	0.944	D (0.001)	D (1.0)	D (1.0)

Key: D—damaging/deleterious, B—benign.

**Table 3:** High-confidence autosomal recessive candidate variants.

Gene	Chr	Exon	cDNA Change	Protein Change	refGene	CADD	REVEL	SIFT	PolyPhen2	Mutation Taster
*CPT2*	1	4	NM_000098:c.T1055G	p.F352C	Nonsynonymous SNV	22.1	0.521	D (0)	D (0.999)	P (0)
*LRP2*	2	69	NM_004525:c.A12628C	p.I4210L	Nonsynonymous SNV	21.8	0.503	D (0)	D (0.995)	P (0)
*ERCC6L2*	9	11	NM_001010895:c.T1742C	p.V581A	Nonsynonymous SNV	20.5	0.512	D (0.001)	-	P (0)
*NXPE1*	11	5	NM_152315:c.G631A	p.G211R	Nonsynonymous SNV	20.2	0.501	D (0)	D (1.0)	P (0.002)

Key: D—damaging/deleterious, P—probably damaging.

**Table 4:** High-confidence autosomal dominant candidate variants.

Gene	Chr	Exon	cDNA Change	Protein Change	refGene	CADD	REVEL	SIFT	PolyPhen2	Mutation Taster
*FOXD4L1*	2	1	NM_01218:c.C433T	p.R145C	Nonsynonymous SNV	23.2	0.666	D (0.008)	B (0.208)	D (1.0)
*HS6ST1*	2	2	NM_004807:c.C745A	p.R249S	Nonsynonymous SNV	28.3	0.827	D (0.001)	D (0.997)	D (1.0)
*HOXD8*	2	1	NM_001199746:c.50A	p.A17D	Nonsynonymous SNV	15.38	0.462	T (0.167)	P (0.634)	D (1.0)
*FOXD4L5*	9	1	NM_001126334:c.G512A	p.R171H	Nonsynonymous SNV	21.8	0.567	D (0)	D (1.0)	D (1.0)
*SVIL*	10	20	NM_003174:c.T2843G	p.L948R	Nonsynonymous SNV	24.4	0.5	D (0.001)	D (0.991)	D (0.995)
*STOX1*	10	1	NM_001130159:c.C163T	p.R55C	Nonsynonymous SNV	26.4	0.535	D (0.001)	D (1.0)	D (0.998)

Key: D—damaging/deleterious, B—benign, P—probably damaging, T—tolerated.

## Data Availability

The raw sequencing data have been deposited in the NCBI Sequence Read Archive (SRA) under accession number PRJNA1270734.

## Supplementary Materials

The following supporting information can be downloaded at https://www.mdpi.com/article/10.3390/genes16101220/s1. Table S1: Detailed genotype analysis of established congenital scoliosis genes.

## Abbreviations

The following abbreviations are used in this manuscript:

CS	Congenital scoliosis
AIS	Adolescent idiopathic scoliosis
WES	Whole-exome sequencing
MZ	Monozygotic twins
KFS	Klippel-Feil syndrome
CSD	Congenital spinal deformity
SNV	Single nucleotide variant
CNV	Copy number variation
VCF	Variant call format
BAM	Binary alignment map
MAF	Minor allele frequency
CADD	Combined annotation dependent depletion
REVEL	Rare exome variant ensemble learner
SIFT	Sorting intolerant from tolerant
HDIV	Human diversity
IGV	Integrative Genomics Viewer
